# Exposure to pesticides and risk of Hodgkin lymphoma in an international consortium of agricultural cohorts (AGRICOH)

**DOI:** 10.1007/s10552-023-01748-1

**Published:** 2023-07-07

**Authors:** Joanne Kim, Maria E. Leon, Leah H. Schinasi, Isabelle Baldi, Pierre Lebailly, Laura E. Beane Freeman, Karl-Christian Nordby, Gilles Ferro, Alain Monnereau, Maartje Brouwer, Kristina Kjaerheim, Jonathan N. Hofmann, Kurt Straif, Hans Kromhout, Joachim Schüz, Kayo Togawa

**Affiliations:** 1 Environment and Lifestyle Epidemiology Branch, International Agency for Research On Cancer, IARC/WHO), Lyon, France; 2 Department of Environmental and Occupational Health, Dornsife School of Public Health, Drexel University, Philadelphia, PA USA; 3 Service Santé Travail Environnement, CHU de Bordeaux, Bordeaux, France; 4 ANTICIPE, INSERM U1086, Université de Caen Normandie, and Centre de Lutte Contre Le Cancer François Baclesse, Caen, France; 5 Occupational and Environmental Epidemiology Branch, Division of Cancer Epidemiology and Genetics, National Cancer Institute (NCI), Bethesda, MD USA; 6 National Institute of Occupational Health (STAMI), Oslo, Norway; 7 Hematological Malignancies Registry of Gironde, Bergonie Institute, Comprehensive Cancer Centre, Bordeaux, France; 8 EPICENE, INSERM U1219, Université de Bordeaux, Bordeaux, France; 9 National Institute for Public Health and the Environment (RIVM), Bilthoven, The Netherlands; 10 Department of Research, Cancer Registry of Norway, Oslo, Norway; 11 Non-communicable Diseases and Environment Programme, IS Global, Barcelona, Spain; 12 Global Observatory On Pollution and Health, Boston College, Chestnut Hill, MA USA; 13 Institute for Risk Assessment Sciences (IRAS), Utrecht University, Utrecht, The Netherlands

**Keywords:** Pesticides, Hodgkin lymphoma, Agricultural exposures, Occupational cancer, Cohort study

## Abstract

**Purpose:**

Some pesticides may increase the risk of certain lymphoid malignancies, but few studies have examined Hodgkin lymphoma (HL). In this exploratory study, we examined associations between agricultural use of 22 individual active ingredients and 13 chemical groups and HL incidence.

**Methods:**

We used data from three agricultural cohorts participating in the AGRICOH consortium: the French Agriculture and Cancer Cohort (2005–2009), Cancer in the Norwegian Agricultural Population (1993–2011), and the US Agricultural Health Study (1993–2011). Lifetime pesticide use was estimated from crop-exposure matrices or self-report. Cohort-specific covariate-adjusted overall and age-specific (< 40 or ≥ 40 years) hazard ratios (HRs) and 95% confidence intervals (CIs) were estimated using Cox regression and combined using random effects meta-analysis.

**Results:**

Among 316 270 farmers (75% male) accumulating 3 574 815 person-years at risk, 91 incident cases of HL occurred. We did not observe statistically significant associations for any of the active ingredients or chemical groups studied. The highest risks of HL overall were observed for the pyrethroids deltamethrin (meta-HR = 1.86, 95% CI 0.76–4.52) and esfenvalerate (1.86, 0.78–4.43), and inverse associations of similar magnitude were observed for parathion and glyphosate. Risk of HL at ≥ 40 years of age was highest for ever-use of dicamba (2.04, 0.93–4.50) and lowest for glyphosate (0.46, 0.20–1.07).

**Conclusion:**

We report the largest prospective investigation of these associations. Nonetheless, low statistical power, a mixture of histological subtypes and a lack of information on tumour EBV status complicate the interpretability of the results. Most HL cases occurred at older ages, thus we could not explore associations with adolescent or young adult HL. Furthermore, estimates may be attenuated due to non-differential exposure misclassification. Future work should aim to extend follow-up and refine both exposure and outcome classification.

**Supplementary Information:**

The online version contains supplementary material available at 10.1007/s10552-023-01748-1.

## Introduction

Hodgkin lymphoma (HL) is a rare cancer diagnosed in an estimated 83 000 individuals each year worldwide [1–3]. In most age groups, HL is slightly more common among males, with overall global age-standardized rates of 1.2 and 0.8 per 100,000 among males and females, respectively [2]. Unlike other lymphomas, its incidence has a bimodal age distribution, with one peak among adolescents and young adults (15–35 years) and another at older ages (50 +) [1]. The tumours are characterized by the presence of malignant Reed-Sternberg cells (1%) of B-cell origin but are otherwise dominated by non-malignant inflammatory and accessory cells [1]. The histological subtypes of HL fall into two main groups, classical and nodular lymphocyte predominant; the vast majority are classical (~ 95%) and therefore most epidemiological data reflects this dominant type [1]. Histologic subtype and Epstein-Barr virus infection (present in 40% of tumours) define epidemiologically and etiologically distinct forms of HL [1, 4]. Known risk factors of EBV-positive classical HL include: family history, genetic polymorphisms in human leukocyte antigen complexes, immune deficiency and smoking, but the causes of EBV-negative classical HL and the much rarer nodular lymphocyte predominant HL are largely unknown [1].

Occupational exposure to pesticides has been suggested as a possible risk factor for HL, with oxidative stress and immunotoxicity suggested as potential mechanisms [5]. However, few studies have evaluated the risk of HL associated with exposure to specific pesticide active ingredients, in part due to the challenges of having sufficient statistical power to study this rare outcome, as well as a lack of data on exposure to specific active ingredients. In a pooled analysis of case–control studies from the USA and Canada, ever use of the organophosphate insecticide terbufos was associated with higher risk of HL overall (odds ratio, OR = 2.58, 95% CI 1.06–6.25), and in age-stratified analyses, additional associations were observed for HL at younger ages (≤ 40 years of age) with the organophosphates dimethoate (OR_age≤40_ = 3.43, 95% CI 1.04–11.34) and malathion (OR_age≤40_ = 1.91, 95% CI 1.07–3.43)[6]. In the Canadian case–control study alone, elevated risks had been reported in association with the phenoxy herbicide dichlorprop (OR = 6.35, 95% CI 1.56–25.92)[7], which was not assessed when pooled with the US studies, as well as with the organophosphate insecticide chlorpyrifos (OR = 5.26, 95% CI 1.56–17.79)[8], which was diminished after pooling (OR = 1.83, 95% CI 0.69–4.89).

To explore associations in a prospective study, including active ingredients not previously investigated, we examined ever vs. never occupational use of 13 pesticide chemical groups and 22 active ingredients in relation to HL incidence in three large agricultural cohorts from France, Norway and the USA participating in the AGRICOH consortium (https://agricoh.iarc.fr/).

## Methods

Details on the study design and exposure assessment were published previously [9, 10] and are described briefly below.

### Study population

#### Agriculture and cancer cohort (AGRICAN), France

Between 2005 and 2007, AGRICAN recruited individuals enrolled in the national health insurance scheme for workers in the agricultural industry (Mutualité Sociale Agricole, MSA) [11]. Eligible men and women were over 18 years of age, had been covered by the MSA for at least 3 years and resided in one of 11 departments in France with population-based cancer registries. The present analysis includes 138 755 active or retired farmers or farm workers, 56% of whom were male; non-farmers were excluded. All participants completed a mailed questionnaire on demographic and lifestyle characteristics as well as lifetime history of agricultural activities such as: crops cultivated, animals raised and use of pesticides on each of 11 different crops, including start and end years. Cohort members were linked to cancer and mortality registries and the National Death Index until 31 December 2009.

#### Cancer in the Norwegian agricultural population (CNAP), Norway

CNAP is an administrative cohort based on Norway’s compulsory agricultural census for farm holders (owners and non-owners operating a farm). Respondents born later than 1924 who had responded to any of the censuses conducted approximately every five years between 1969 and 1989 were included in the cohort. Linkage across censuses and to other population registries was facilitated by the unique personal identification number assigned to all residents of Norway. A total of 147 134 farm holders were identified, 84% of whom were male [12]. For the year preceding the census, respondents reported their crop and livestock production, acreage and production technology. In certain years, farmers were also asked to report their purchases of pesticides (1969) and the type of spraying equipment on the farm (1979). Responses from the agricultural censuses conducted between 1969 and 1989, as well as those in 1999 and 2010, were linked to each cohort member. Linkage with the national cancer and mortality registries was conducted up until 31 December 2011.

#### Agricultural health study (AHS), USA

The AHS recruited individuals applying for or renewing a restricted-use pesticide license in Iowa and North Carolina between 1993 and 1997 [13]. The vast majority (97%) of the 52 394 private pesticide applicators were males who either owned or worked on a farm; commercial pesticide applicators were excluded from this analysis. Participants completed a questionnaire on demographic and lifestyle characteristics, agricultural activities and their use of over 50 individual pesticide active ingredients including the duration, frequency and decade of first use. Participants also reported information on pesticide application practices, including the type of spraying equipment and personal protective equipment used. Two-thirds (66%) completed a follow-up questionnaire on pesticide use five years later [14]; among non-respondents, multiple imputation was used to impute missing information on pesticide use since enrolment [15]. Imputation models included variables such as demographic (age, sex, state, county, marital status, education), farm (ownership, size, crops) and pesticide use characteristics (years and days/year mixing, application method, application uses, gloves), as well as self-reported chronic conditions; more details provided here [15]. Cohort members were linked to the National Death Index and the state cancer and mortality registries from enrollment until 31 December 2011 in Iowa and 31 December 2010 in North Carolina.

#### Ethics approval

Each cohort study received ethics approval from their respective institutions. These included Statistics Norway for CNAP and the National Cancer Institute for AHS. AGRICAN was approved by the Advisory Committee on Information Processing in Material Research in the field of Health (Comité Consultatif sur le Traitement de l’Information en matière de Recherche dans le domaine de la Santé, number 01.148) and by the French data protection authority (Commission Nationale Informatique et Libertés, number 05.1292). This pooling project was also approved by the International Agency for Research on Cancer Ethics Committee (Project 12–28) (Table 1).

**Table 1:** Description of the study population

	Combined population		AGRICAN		CNAP		AHS	
	*n*	%	*n*	%	*n*	%	*n*	%
Total	316 270	100	127 282	100	137 821	100	51 167	100
Males	237 317	75	71 358	56	116 128	84	49 831	97
Females	78 953	25	55 924	44	21 693	16	1336	3
Ever smoker (% of non-missing)	65 208	39	41 980	35			23 228	47
Ever use of at least one pesticide active ingredient or chemical group	198 492	63	80 898	67	62 047	45	51 542	99
Organophosphate Insecticides	185 950	59	80 943	64	57 593	42	47 414	93
Chlorpyrifos	94 038	30	72 429	57	n.a	n.a	21 609	42
Malathion	144 629	46	51 696	41	56 717	41	36 216	71
Parathion	136 643	43	73 460	58	54 623	40	8 560	17
Carbamate Insecticides	168 447	53	80 853	64	52 408	38	35 186	69
Organochlorine Insecticides	162 964	52	82 299	65	53 126	39	27 539	54
DDT	108 784	34	57 434	45	37 851	27	13 499	26
Lindane	137 161	43	79 826	63	47 267	34	10 068	20
Pyrethroid Insecticides	130 611	41	66 652	52	49 668	36	14 291	28
Deltamethrin	99 584	31	65 542	51	34 026	25	16	0
Esfenvalerate	85 692	27	53 128	42	32 061	23	503	1
Permethrin	103 751	33	45 749	36	49 668	36	8 334	16
Other herbicide: Dicamba	103 577	33	42 224	33	34 656	25	26 697	52
Other herbicide: Glyphosate	140 318	44	46 147	36	51 928	38	42 243	83
Ever engaged in animal production	242 695	77	107 505	84	102 578	74	32 612	64
Year of birth (range)	1900–1985		1900–1985		1925–1971		1901–1983	
Follow-up period (range)	1993–2011		2005–2009		1993–2011		1993–2011	
Median age at start of follow-up, years	55		67		51		46	
Median duration of follow-up, years	16		4		19		16	
Person-years of follow-up	3 574 815		426 340		2 396 595		751 880	
Incident Hodgkin lymphomas (HL)	91		15		57		19	
*Subtype:* Classical HL	80		15		47		18	
*Subtype:* Nodular lymphocyte predominant HL	11		0		10		1	
Median age at diagnosis, years (range)	58 (26 to 88)		72 (30 to 88)		58 (35 to 78)		43 (26 to 74)	

*AGRICAN* agriculture and cancer cohort (France); *CNAP* cancer in the Norwegian population (Norway); *AHS* agricultural health study (USA)

### Selection of active ingredients

The active ingredients to be investigated were selected based on the following criteria: used in at least two of the three countries and with some mechanistic, animal, or human evidence for an association with lymphohaematological malignancies; frequently used chemicals not previously investigated in epidemiological studies were also selected. This resulted in a list of 33 active ingredients belonging to 14 chemical groups (organophosphate, organochlorine, carbamate and pyrethroid insecticides; phenyl urea, chloroacetanilide, dinitroaniline, phenoxy, thiocarbamate, triazine and triazinone herbicides; dithiocarbamate and phthalimide fungicides; and arsenical pesticides). However, we report results only for the 22 active ingredients and 13 chemical groups for which at least two cohorts had at least 5 exposed cases each (see Table 2).

**Table 2:** Meta-hazard ratios (meta-HRs) for ever use of 13 pesticide chemical groups and 22 active ingredients and incidence of Hodgkin lymphoma in three agricultural cohorts from France, Norway, and the USA

	Hodgkin lymphoma, overall					Hodgkin lymphoma, diagnosed at ≥ 40 years			
	N_exp_	meta-HR^a^	95% CI	I^2^		N_exp_	meta-HR^a^	95% CI	I^2^
Organophosphate Insecticides	50	0.64	0.22–1.90	0%		41	0.56	0.15–2.12	0%
Chlorpyrifos	16	0.81^b^	0.34–1.91	0%					
Malathion	41	0.62	0.18–2.11	49%		34	0.59	0.23–1.53	0%
Parathion	29	0.53^c^	0.17–1.66	0%		28	0.68^c^	0.18–2.54	0%
Carbamate Insecticides	44	1.08	0.47–2.44	0%		39	1.49	0.59–3.71	0%
Aldicarb	18	1.23^c^	0.53–2.84	0%		16	1.10^c^	0.46–2.62	0%
Carbaryl	17	0.64^b^	0.22–1.83	21%		15	0.80^b^	0.28–2.28	0%
Pirimicarb	28	0.81^c^	0.26–2.48	0%		26	0.94^c^	0.29–3.00	0%
Organochlorine Insecticides	41	1.29	0.60–2.76	17%		35	0.90	0.40–2.05	0%
DDT	27	1.79^c^	0.73–4.37	0%		27	1.95^c^	0.73–5.18	0%
Lindane	35	1.40	0.63–3.09	27%		28	1.15^c^	0.44–3.02	0%
Pyrethroid Insecticides	35	1.18	0.51–2.74	0%					
Deltamethrin	25	1.86^c^	0.76–4.52	3%		22	1.74^c^	0.70–4.35	0%
Esfenvalerate	22	1.86^c^	0.78–4.43	0%		19	1.71^c^	0.69–4.25	0%
Permethrin	26	1.25^c^	0.41–3.77	0%		24	1.35^c^	0.43–4.28	0%
(Phenyl) Urea Herbicides	32	0.89^c^	0.32–2.48	0%		29	0.84^c^	0.28–2.53	0%
Linuron	30	0.78^c^	0.27–2.28	0%		28	0.85^c^	0.27–2.65	0%
Chloroacetanilide Herbicides	25	1.18^d^	0.40–3.46	47%		20	1.59^d^	0.66–3.86	1%
Dinitroaniline Herbicides	19	0.71^b^	0.19–2.63	41%		14	0.60^b^	0.15–2.43	35%
Phenoxy Herbicides	45	1.01	0.44–2.29	0%		39	1.51	0.55–4.17	0%
2,4-D	44	1.25	0.55–2.84	0%		38	1.83	0.66–5.08	0%
MCPA	28	1.54^c^	0.46–5.11	0%		26	1.49^c^	0.43–5.19	0%
MCPP	27	1.42^c^	0.46–4.38	0%		25	1.40^c^	0.44–4.48	0%
Thiocarbamate Herbicides	18	0.98^b^	0.40–2.36	0%		13	0.79^b^	0.28–2.20	0%
EPTC	24	1.66^d^	0.73–3.80	0%					
Triazine Herbicides	45	1.05	0.44–2.48	0%		36	0.91	0.35–2.38	0%
Atrazine	20	0.61^b^	0.23–1.62	0%		15	0.69^b^	0.21–2.22	0%
Triazinone Herbicides	34	0.91	0.43–1.93	0%		30	0.97	0.41–2.29	0%
Metribuzin	34	0.91	0.43–1.94	0%		30	0.97	0.41–2.29	0%
Other Herbicides									
Dicamba	35	1.63	0.83–3.22	0%		31	2.04	0.93–4.50	0%
Glyphosate	40	0.58	0.29–1.18	0%		26	0.46^d^	0.20–1.07	0%
Dithiocarbamate Fungicides	33	1.22^c^	0.46–3.26	0%		30	1.14^c^	0.40–3.26	0%
Mancozeb	33	1.21^c^	0.47–3.15	0%		30	1.13^c^	0.40–3.15	0%
Phthalimide Fungicides	29	0.58^c^	0.17–1.94	0%		27	0.72^c^	0.17–3.03	0%
Captafol	25	0.68^c^	0.26–1.78	0%		24	0.70^c^	0.24–2.01	0%

Only results based on at least two cohorts with 5 or more exposed cases each are reported. Pesticides that were investigated but did not meet the reporting rules were: arsenical pesticides and the active ingredients dichlorvos, terbufos, carbofuran, isoproturon, alachlor, metolachlor, trifluralin, butylate, simazine, thiram, and captan

Abbreviations: meta-HR, meta-hazard ratio; CI, confidence interval; N_exp_, number of exposed cases; p_trend,_ p-value for trend; DDT, dichlorodiphenyltrichloroethane; 2,4-D, 2,4-dichlorophenoxyacetic acid; MCPA, 2-methyl-4-chlorophenoxyacetic acid; MCPP, methylchlorophenoxypropionic acid; EPTC, S-ethyl dipropylthiocarbamate

^a^meta-HRs combining cohort-specific HR estimates adjusted for cohort-specific sets of confounders. AGRICAN: Cox regression adjusted for sex, livestock, retirement status, number of selected types of crops for which pesticide treatment personally applied, smoking status (current, former, or never); CNAP: Cox regression adjusted for sex, livestock, dichlorvos, aldicarb, lindane, DDT, deltamethrin, mancozeb, linuron, glyphosate; AHS: Cox regression adjusted for sex, state, livestock, terbufos, lindane, DDT, permethrin, dicamba, parathion, carbaryl, smoking status (current, former, or never)

^b^Meta-analysis based on AGRICAN and AHS only (9 estimates)

^c^Meta-analysis based on AGRICAN and CNAP only (31 estimates)

^d^Meta-analysis based on CNAP and AHS only (4 estimates)

### Assessment of exposure to specific active ingredients

For AGRICAN and CNAP, country- and year- specific crop-exposure matrices assigning exposure to the selected active ingredients were developed based on registration and sales data from France and Norway, as well as their recommended use [9, 16]. These crop-exposure matrices were combined with each participant’s lifetime history of crop production and whether they reported using pesticides on specific crops (AGRICAN), or purchasing pesticides or owning spraying equipment (CNAP), to estimate exposure status (yes/no) to each active ingredient in any given year from 1950 until the last year of cancer follow-up [9]. For participants in the AHS, exposure status was based on self-reported use of each active ingredient in the baseline and follow-up questionnaires. This information was then used to assess whether each participant was ever exposed and the duration of their exposure to each active ingredient studied, which were the only exposure metrics available across all three cohorts.

### Follow-up and cancer ascertainment

The outcome of interest was the first incident HL during follow-up, identified using International Classification of Diseases for Oncology third edition (ICD-O-3) morphology codes and the proposed subtype groupings of the International Lymphoma Epidemiology Consortium [17]: 9650–9655 and 9661–9667 for classical HL and 9659 for nodular lymphocyte predominant HL. Tumour EBV infection status was not captured in the registries. Follow-up began at the date of enrolment (AGRICAN, AHS), or 1 January 1993 (CNAP) which was the earliest year of follow-up in the other cohorts. Participants were censored at the earliest date of: (i) first incident cancer (except non-melanoma skin cancer, which is not well-captured in cancer registries); (ii) loss to follow-up or migration out of the cancer registry area; (iii) death; or (iv) end of follow-up. Participants with any prevalent cancer (except non-melanoma skin cancer) were excluded from this analysis.

### Statistical analysis

We used Cox regression models to estimate cohort-specific hazard ratios (HRs) and 95% confidence intervals (CIs) for incident HL in relation to ever use of each active ingredient and chemical group. In addition, we ran secondary analyses by duration of use (< or ≥ 16 years, the median duration), including a test for linear trend across the categories, and for age-specific incident HL (< 40 and ≥ 40 years, separately) due to the heterogeneity of HL by age at diagnosis. In all models, the reference group consisted of never-users of the given pesticide or chemical group, and we used age as the time scale and adjusted for sex and animal production. Additional cohort-specific covariates were: retirement status (AGRICAN); state of residence (AHS); and smoking status (AGRICAN, AHS). In addition, as a proxy for additional pesticide exposure, the number of crops personally treated with pesticides was adjusted for in AGRICAN; to capture similar information, a set of specific active ingredients was included in models for CNAP and AHS (see Table 2 footnote). All variables were modelled as time-fixed.

When available from at least two cohorts with ≥ 5 exposed cases each, the fully-adjusted cohort-specific HR estimates were combined using random effects meta-analysis. We therefore report meta-HRs associated with the use of 22 active ingredients and 13 chemical groups. Not all meta-HRs in the age-specific and exposure duration analyses met the reporting criteria. The I^2^ statistic was used to assess heterogeneity for all meta-HRs. All analyses were conducted using Stata 14.

## Results

Across the three cohorts, a total of 316 270 farmers (127 282 AGRICAN, 137 821 CNAP and 51 167 AHS; Supplementary Fig. 1) contributed 3 574 815 person-years. The median follow-up duration was 16 years overall, ranging from 4 years in AGRICAN to 19 years in CNAP (Table 1). Most participants were male (75%), but the proportion of males varied between cohorts from 56% in AGRICAN to 84% in CNAP and 97% in AHS. The median age at the start of follow-up was 46 years in AHS, 51 years in CNAP and 67 years in AGRICAN. The proportion of current or former smokers was 35% in AGRICAN and 47% in AHS; information on smoking status was not available in CNAP. We estimated that fewer than half of the farm owners in the CNAP cohort had ever used one of the selected active ingredients or chemical groups (45%), while a greater proportion of AGRICAN farmers (67%) and almost all AHS private applicators (99%) were ever users (Table 1). Additional details on the characteristics of the study participants [10] and their exposure to pesticides [9] have been published.

Among the various chemical classes evaluated, organophosphate insecticides were the most prevalent (used by 59% of farmers), followed by carbamate and organochlorine insecticides (53% and 52% of farmers, respectively; Table 1). The most prevalent active ingredients were: the organophosphate insecticide malathion (used by 46% of farmers), the phenoxy herbicide 2,4-D (45%) and glyphosate (45%). Phenoxy herbicides were used the longest (e.g. 2,4-D was used for a median of 24 years, range: 1 to 56 years) whereas newer pesticides such as pyrethroids were used for a shorter duration, with less variability estimated between farmers (e.g. deltamethrin was used for a median of 9 years, range: 1 to 31 years). The estimated exposure prevalence and duration of use of each active ingredient and chemical group can be found in Supplementary Table 1 and in more detail in Brouwer et al. 2016 Supplementary Table S5 [9].

In the combined population, a total of 91 incident HLs were observed, of which 80 (88%) were classical and the remaining 11 (12%) were nodular lymphocyte predominant. The median age at diagnosis was 58 years (range: 26 to 88 years), though this varied from 43 years in AHS to 72 years in AGRICAN, reflecting differences in median age at the start of follow-up in each cohort (Table 1). Only 14 HL cases occurred before the age of 40 years.

We did not observe any statistically significant associations between the 22 active ingredients or 13 chemical groups examined and the risk of HL (Table 2). The highest risks of HL overall were observed among ever users of the pyrethroid insecticides deltamethrin and esfenvalerate, with meta-HRs (and 95% CIs) of 1.86 (0.76–4.52) and 1.86 (0.78–4.43), respectively. Inverse associations of similar magnitude were observed for the organophosphate insecticide parathion (0.53, 0.17–1.66) and the broad-spectrum herbicide glyphosate (0.58, 0.29–1.18). In general, compared to the active ingredients, the meta-HRs for the 13 chemical groups were closer to the null, with point estimates ranging from 1.01 to 1.29 for positive associations and from 0.64 to 0.98 for inverse associations (Table 2). For most meta-estimates, we did not observe evidence of heterogeneity, with a few exceptions (malathion, chloroacetanilide and dinitroaniline herbicides, I^2^ = 41–49%).

In secondary analyses, the risk of HL diagnosed at ≥ 40 years of age was two-fold in association with dicamba (meta-HR_age≥40_ = 2.04, 95% CI 0.93–4.50) and inversely associated with glyphosate (0.46, 0.20–1.07); all confidence intervals crossed the null. There were too few exposed HL cases younger than 40 to report HRs for this outcome in relation to any of the active ingredients or chemical groups examined. Among the few instances in which there was a sufficient number of exposed cases in each category of exposure duration (< or ≥ 16 years), no associations or linear trends were observed (Supplementary Table 2).

## Discussion

In this exploratory prospective analysis of three agricultural cohorts, we did not observe statistically significant associations between any of the 22 active ingredients or 13 chemical groups and the risk of HL. We observed some slightly elevated and some slightly diminished hazard ratios with wide confidence intervals that crossed the null. The highest risks of HL overall were observed for the pyrethroids deltamethrin and esfenvalerate, and inverse associations of similar magnitude were observed for parathion and glyphosate. Farmers who had ever used dicamba had approximately two-fold higher risk of developing HL at ≥ 40 years of age. While mechanistic evidence for these pesticides as potential carcinogens is moderate (dicamba) [18–25] to strong (pyrethroids) [26], there have been few epidemiological investigations, and none have been conclusive [6, 27]. To our knowledge, ours is the first epidemiological study investigating associations between synthetic pyrethroids (permethrin, deltamethrin, and esfenvalerate) and the risk of HL.

The low incidence of HL and prevalence of specific active ingredients contributed to the low precision of our estimates, posing challenges for reporting and comparing results. We could not examine previously reported positive findings in the North American pooled case–control studies due to not having assessed exposure to the certain active ingredients (dichlorprop and dimethoate) or not having a sufficient number of cases exposed to terbufos or cases under 40 years exposed to malathion [6, 7]. A hospital-based French case–control study had reported positive associations between HL and use of chemical groups we did not assess (pyrethrin insecticides, triazole fungicides, and phenoline, picoline and amide herbicides), as well as with groups not associated with HL in our analysis (organochlorine insecticides, carbamate fungicides, and urea herbicides) [28]. However, most of these previously reported elevated risks were based on relatively few exposed cases (4 to 8), with the exception of malathion, which was associated with HL at ≤ 40 years in the North American Pooled Project based on 26 exposed cases [6].

Differences in the age distribution between studies and underlying etiological differences for HL by age may explain some inconsistencies between our findings and the extant literature, therefore reporting age-stratified results may facilitate comparability between studies. Examining risk factors for HL by histological subtype and tumour EBV status is preferable, but the rare nature of this cancer and lack of EBV status information have hindered such analyses in our study and in previous studies. However, some studies used age as a proxy, since the proportion of EBV + tumours is slightly higher among older adults than among younger adults [4].

In this exploratory analysis, we estimated a large number of associations and therefore cannot rule out that some of the suggestive positive or inverse findings occurred simply by chance; thus, our results should be interpreted with caution. Since semi-quantitative exposure information (e.g. probability, frequency) was available in only one of the three cohorts (AHS), we reported results by ever vs. never and duration of use. However, ever vs. never represents a meaningful exposure contrast among farmers, since they are exposed at higher levels than the general population and tend to use a particular active ingredient for several years. Follow-up times varied between cohorts as well as across specific pesticides, since their use has changed over time (for example, due to pesticide bans and replacements). The prevalence of pesticide use overall and of specific active ingredients varied between cohorts (Supplementary Table 1) due to the different recruitment strategies and predominant crops of each country. The different recruitment strategies combined with the gendered nature of farm ownership and farming tasks also led to different proportions of females in each cohort. Despite these differences, we did not find much evidence of statistical heterogeneity between cohorts in the meta-estimates; however, like other indices of heterogeneity, the I^2^ statistic is biased when the number of meta-analysed studies is small [29]. Furthermore, non-differential exposure misclassification, particularly from the use of crop-exposure matrices, may have biased our estimates towards the null [9]. Refinement of exposure assessment is ongoing, including the addition of probability and frequency estimates and consideration of exposure through tasks other than pesticide application (e.g. crop picking). Since men are more likely to apply pesticides than female farmers, who tend to be exposed to pesticides through contact with recently-treated crops, this will reduce misclassification and account for differences in exposure patterns between male and female farmers.

Despite these limitations, this analysis represents the best available data assessing the relationship between exposure to specific pesticides and the risk of HL. The prospective nature of the data avoids recall bias, which may have affected previous findings from case–control studies. Since the analysis is restricted to farmers, it overcomes bias from the lower mortality and cancer incidence commonly observed among farmers compared to the general population, often attributed to their lower smoking rates and possibly higher levels of physical activity [30]. We also made efforts to adjust for exposure to other pesticides, by controlling for individual active ingredients as well as animal production, which likely involves pesticide use. However, we cannot rule out residual confounding due to unmeasured potential confounders, such as EBV infection and genetic predisposition, though there is no reason to believe these would be correlated with occupational use of specific pesticides. Furthermore, most HL cases occurred at older ages, thus we could not explore associations with adolescent or young adult HL. Future work using larger databases with even longer years of follow-up is needed to investigate these associations further, with more refined exposure assessment methods, and, if possible, ascertainment of tumour EBV status, histological subtype, and greater numbers of younger HL cases.

### Supplementary Information

Below is the link to the electronic supplementary material.

Supplementary file1 (DOCX 57 KB)

## Data Availability

Data can be made available upon reasonable request to the corresponding author. Data from the AHS can be requested through this website: https://aghealth.nih.gov/collaboration/process.html.
