# Supplementary material for: Exposure to pesticides and risk of Hodgkin lymphoma in an international consortium of agricultural cohorts (AGRICOH)
Source: Cancer Causes Control. 2023 Jul 7;34(11):995–1003. doi: 10.1007/s10552-023-01748-1 (PMC10533587; doi:10.1007/s10552-023-01748-1)
Supplement: Supplementary file 1 — Supplementary file1 (DOCX 57 KB) [file 10552_2023_1748_MOESM1_ESM.docx]

## Supplementary Material

**Exposure to pesticides and risk of Hodgkin lymphoma in an international consortium of agricultural cohorts (AGRICOH)**

Joanne Kim^1^, Maria E. Leon^1^, Leah H. Schinasi^2^, Isabelle Baldi^3^, Pierre Lebailly^4^, Laura E. Beane Freeman^5^, Karl-Christian Nordby^6^, Gilles Ferro^1^, Alain Monnereau^7,8^, Maartje Brouwer^9^, Kristina Kjaerheim^10^, Jonathan N. Hofmann^5^, Kurt Straif^11,12^, Hans Kromhout^13^, Joachim Schüz^1^, Kayo Togawa^1^

^1^Environment and Lifestyle Epidemiology Branch, International Agency for Research on Cancer, Lyon (IARC/WHO), France

^2^Department of Environmental and Occupational Health, Dornsife School of Public Health, Drexel University, Philadelphia, PA, USA

^3^Service Santé Travail Environnement, CHU de Bordeaux, Bordeaux, France

^4^ANTICIPE, U1086 INSERM, Université de Caen Normandie, and Centre de Lutte Contre le Cancer François Baclesse, Caen, France

^5^Occupational and Environmental Epidemiology Branch, Division of

Cancer Epidemiology and Genetics, National Cancer Institute (NCI), Bethesda, MD, USA

^6^National Institute of Occupational Health (STAMI), Oslo, Norway

^7^Hematological Malignancies Registry of Gironde, Bergonie Institute, Comprehensive Cancer Centre, Bordeaux, France

^8^EPICENE, U1219 INSERM, Université de Bordeaux, Bordeaux, France

^9^National Institute for Public Health and the Environment (RIVM), Bilthoven, the Netherlands

^10^Department of Research, Cancer Registry of Norway, Oslo, Norway

^11^Non-communicable Diseases and Environment Programme, IS Global, Barcelona, Spain

^12^Global Observatory on Pollution and Health, Boston College, Chestnut Hill, MA, USA

^13^Institute for Risk Assessment Sciences (IRAS), Utrecht University, Utrecht, the Netherlands

**Corresponding author:**

Joachim Schüz

150 Cours Albert Thomas

69372 Lyon CEDEX 08

France

Tel: +33 (0)4 72 73 84 85

Fax: +33 (0)4 72 73 85 75

**Supplementary Figure 1.** Cohort-specific exclusions and the resulting study populations included in the meta-analysis (adapted from Leon et al. 2019)

*Excluded population*

Reference: Leon ME, Schinasi LH, Lebailly P, Beane Freeman LE, Nordby KC, Ferro G*, et al.* Pesticide use and risk of non-Hodgkin lymphoid malignancies in agricultural cohorts from France, Norway and the USA: a pooled analysis from the AGRICOH consortium. Int J Epidemiol **2019**;48(5):1519-35 doi 10.1093/ije/dyz017.

**Supplementary Table 1.** Estimated exposure prevalence and duration (median and range, in years) for each selected active ingredient and chemical group (adapted from Brouwer et al. 2016 Supplementary Table S5)

|  | Combined population | | | AGRICAN | | | CNAP | | | AHS | | |
| --- | --- | --- | --- | --- | --- | --- | --- | --- | --- | --- | --- | --- |
|  | n | % | Duration | n | % | Duration | n | % | Duration | n | % | Duration |
| Organophosphate Insecticides | 185 950 | 59% | 23 (1, 58) | 80 943 | 64% | 26 (1, 58) | 57 593 | 42% | 25 (1, 45) | 47 414 | 93% | 16 (1, 56) |
| Chlorpyrifos | 94 038 | 30% | 16 (1, 38) | 72 429 | 57% | 19 (1, 36) | n.a. | n.a. | n.a. | 21 609 | 42% | 5 (1, 38) |
| *Dichlorvos* | 77 834 | 25% | 16 (1, 46) | 49 315 | 39% | 20 (1, 42) | 23 719 | 17% | 10 (1, 30) | 4 800 | 9% | 9 (2, 46) |
| Malathion | 144 629 | 46% | 18 (1, 52) | 51 696 | 41% | 25 (1, 52) | 56 717 | 41% | 20 (1, 34) | 36 216 | 71% | 9 (1, 47) |
| Parathion | 136 643 | 43% | 19 (1, 54) | 73 460 | 58% | 19 (1, 54) | 54 623 | 40% | 20 (1, 43) | 8 560 | 17% | 4 (1, 47) |
| *Terbufos* | 46 181 | 15% | 11 (1, 36) | 27 066 | 21% | 15 (1, 27) | n.a. | n.a. | n.a. | 19 115 | 37% | 9 (1, 36) |
| Carbamate Insecticides | 168 447 | 53% | 20 (1, 52) | 80 853 | 64% | 25 (1, 50) | 52 408 | 38% | 20 (1, 26) | 35 186 | 69% | 9 (1, 52) |
| Aldicarb | 80 635 | 25% | 15 (1, 38) | 50 207 | 39% | 21 (1, 38) | 23 719 | 17% | 10 (1, 21) | 6 709 | 13% | 4 (1, 31) |
| Carbaryl | 115 590 | 37% | 19 (1, 49) | 80 617 | 63% | 25 (1, 49) | 5 215 | 4% | 10 (1, 15) | 29 758 | 58% | 9 (1, 43) |
| *Carbofuran* | 42 039 | 13% | 15 (1, 36) | 28 492 | 22% | 19 (1, 36) | n.a. | n.a. | n.a. | 13 547 | 26% | 4 (1, 36) |
| Pirimicarb | 111 113 | 35% | 20 (1, 38) | 60 276 | 47% | 19 (1, 38) | 50 837 | 37% | 20 (1, 20) | n.a. | n.a. | n.a. |
| Organochlorine Insecticides | 162 964 | 52% | 22 (1, 58) | 82 299 | 65% | 27 (1, 58) | 53 126 | 39% | 22 (1, 45) | 27 539 | 54% | 8 (1, 52) |
| DDT | 108 784 | 34% | 13 (1, 35) | 57 434 | 45% | 15 (1, 26) | 37 851 | 27% | 15 (1, 22) | 13 499 | 26% | 4 (1, 35) |
| Lindane | 137 161 | 43% | 22 (1, 47) | 79 826 | 63% | 24 (1, 47) | 47 267 | 34% | 22 (1, 42) | 10 068 | 20% | 4 (1, 46) |
| Pyrethroid Insecticides | 130 611 | 41% | 15 (1, 35) | 66 652 | 52% | 18 (1, 35) | 49 668 | 36% | 15 (1, 15) | 14 291 | 28% | 5 (1, 33) |
| Deltamethrin | 99 584 | 31% | 9 (1, 31) | 65 542 | 51% | 16 (1, 31) | 34 026 | 25% | 9 (6, 9) | 16 | 0% | 5 (3, 8) |
| Esfenvalerate | 85 692 | 27% | 5 (1, 21) | 53 128 | 42% | 12 (1, 21) | 32 061 | 23% | 2 (1, 2) | 503 | 1% | 5 (1, 9) |
| Permethrin | 103 751 | 33% | 15 (1, 33) | 45 749 | 36% | 15 (1, 26) | 49 668 | 36% | 15 (1, 15) | 8 334 | 16% | 4 (1, 33) |
| (Phenyl) Urea Herbicides | 138 932 | 44% | 21 (1, 49) | 77 434 | 61% | 23 (1, 49) | 52 937 | 38% | 21 (1, 31) | 8 561 | 17% | 5 (1, 9) |
| Isoproturon | 60 881 | 19% | 11 (1, 36) | 31 547 | 25% | 19 (1, 36) | 29 334 | 21% | 11 (1, 11) | n.a. | n.a. | n.a. |
| Linuron | 134 845 | 43% | 21 (1, 42) | 75 292 | 59% | 22 (1, 42) | 52 937 | 38% | 21 (1, 31) | 6 616 | 13% | 5 (2, 8) |
| Chloroacetanilide Herbicides | 91 053 | 29% | 10 (1, 43) | 28 830 | 23% | 21 (1, 40) | 23 753 | 17% | 10 (6, 10) | 38 470 | 75% | 13 (1, 43) |
| *Alachlor* | 56 849 | 18% | 16 (1, 40) | 28 830 | 23% | 21 (1, 40) | n.a. | n.a. | n.a. | 28 019 | 55% | 9 (1, 36) |
| *Metolachlor* | 55 877 | 18% | 13 (1, 36) | 27 715 | 22% | 17 (1, 32) | n.a. | n.a. | n.a. | 28 162 | 55% | 9 (1, 36) |
| Dinitroaniline Herbicides | 83 958 | 27% | 16 (1, 46) | 47 675 | 37% | 22 (1, 42) | n.a. | n.a. | n.a. | 36 283 | 71% | 11 (1, 46) |
| *Trifluralin* | 58 667 | 19% | 16 (1, 42) | 32 578 | 26% | 22 (1, 42) | n.a. | n.a. | n.a. | 26 089 | 51% | 9 (1, 39) |
| Phenoxy Herbicides | 145 609 | 46% | 25 (1, 57) | 48 608 | 38% | 26 (1, 56) | 57 167 | 41% | 26 (1, 45) | 39 834 | 78% | 16 (1, 57) |
| 2,4-D | 141 465 | 45% | 24 (1, 56) | 48 608 | 38% | 26 (1, 56) | 54 249 | 39% | 27 (1, 45) | 38 608 | 75% | 16 (1, 48) |
| MCPA | 96 883 | 31% | 26 (1, 55) | 40 918 | 32% | 26 (1, 55) | 55 922 | 41% | 27 (1, 45) | 43 | 0% | 5 (2, 6) |
| MCPP | 94 585 | 30% | 24 (1, 49) | 38 111 | 30% | 22 (1, 49) | 55 922 | 41% | 27 (1, 40) | 552 | 1% | 5 (1, 9) |
| Thiocarbamate Herbicides | 138 536 | 44% | 19 (1, 52) | 65 848 | 52% | 20 (1, 46) | 48 377 | 35% | 20 (1, 29) | 24 311 | 48% | 4 (1, 52) |
| *Butylate* | 41 735 | 13% | 9 (1, 28) | 24 709 | 19% | 13 (1, 15) | n.a. | n.a. | n.a. | 17 026 | 33% | 4 (1, 28) |
| EPTC | 72 874 | 23% | 11 (1, 42) | 27 944 | 22% | 17 (1, 28) | 31 288 | 23% | 10 (1, 20) | 13 642 | 27% | 4 (1, 42) |
| Triazine Herbicides | 159 990 | 51% | 19 (1, 53) | 74 145 | 58% | 22 (1, 47) | 44 187 | 32% | 20 (1, 33) | 41 658 | 81% | 16 (1, 53) |
| Atrazine | 85 184 | 27% | 17 (1, 45) | 45 555 | 36% | 22 (1, 43) | n.a. | n.a. | n.a. | 39 629 | 77% | 16 (1, 45) |
| *Simazine* | 62 965 | 20% | 22 (1, 43) | 50 930 | 40% | 23 (1, 43) | 7 683 | 6% | 13 (1, 33) | 4 352 | 9% | 5 (1, 9) |
| Triazinone Herbicides | 126 458 | 40% | 11 (1, 37) | 60 729 | 48% | 19 (1, 37) | 43 458 | 32% | 11 (1, 21) | 22 271 | 44% | 4 (1, 29) |
| Metribuzin | 126 442 | 40% | 11 (1, 37) | 60 729 | 48% | 19 (1, 37) | 43 458 | 32% | 11 (1, 21) | 22 255 | 43% | 4 (1, 29) |
| Other Herbicides |  |  |  |  |  |  |  |  |  |  |  |  |
| Dicamba | 103 577 | 33% | 18 (1, 44) | 42 224 | 33% | 23 (1, 44) | 34 656 | 25% | 20 (1, 28) | 26 697 | 52% | 9 (1, 36) |
| Glyphosate | 140 318 | 44% | 16 (1, 36) | 46 147 | 36% | 18 (1, 33) | 51 928 | 38% | 20 (1, 20) | 42 243 | 83% | 9 (1, 36) |
|  |  |  |  |  |  |  |  |  |  | (continued on next page) | | |
| Dithiocarbamate Fungicides | 139 281 | 44% | 21 (1, 56) | 81 985 | 64% | 26 (1, 56) | 50 904 | 37% | 19 (1, 45) | 6 392 | 12% | 5 (1, 41) |
| Mancozeb | 135 353 | 43% | 21 (1, 46) | 79 244 | 62% | 24 (1, 46) | 50 904 | 37% | 18 (1, 31) | 5 205 | 10% | 4 (1, 40) |
| *Thiram* | 59 540 | 19% | 24 (1, 53) | 51 778 | 41% | 25 (1, 53) | 7 683 | 6% | 18 (1, 43) | 79 | 0% | 5 (4, 7) |
| Phthalimide Fungicides | 131 267 | 42% | 17 (1, 54) | 76 235 | 60% | 22 (1, 54) | 49 023 | 36% | 15 (1, 35) | 6 009 | 12% | 1 (1, 47) |
| Captafol | 113 810 | 36% | 17 (1, 28) | 70 649 | 56% | 19 (1, 28) | 43 155 | 31% | 10 (1, 17) | n.a. | n.a. | n.a. |
| *Captan* | 62 384 | 20% | 16 (1, 54) | 29 775 | 23% | 22 (1, 54) | 26 713 | 19% | 13 (1, 35) | 5 896 | 12% | 1 (1, 47) |
| *Arsenical Pesticides* | 60 165 | 19% | 18 (1, 53) | 58 047 | 46% | 18 (1, 53) | n.a. | n.a. | n.a. | 2 118 | 4% | 6 (1, 9) |

Abbreviations: AGRICAN, Agriculture and Cancer Cohort (France); CNAP, Cancer in the Norwegian Population (Norway); AHS, Agricultural Health Study (USA); DDT, dichlorodiphenyltrichloroethane; 2,4-D, 2,4-dichlorophenoxyacetic acid; MCPA, 2-methyl-4-chlorophenoxyacetic acid; MCPP, methylchlorophenoxypropionic acid; EPTC, S-ethyl dipropylthiocarbamate; n.a., not assessed

Note: Active ingredients and chemicals groups in italics indicate those for which exposure was assessed but the number of exposed cases did not meet our reporting criteria.

Reference: Brouwer M, Schinasi L, Beane Freeman LE, Baldi I, Lebailly P, Ferro G, Nordby KC, Schuz J, Leon ME, Kromhout H. Assessment of occupational exposure to pesticides in a pooled analysis of agricultural cohorts within the AGRICOH consortium. Occup Environ Med 2016;73: 359-67.

**Supplementary Table 2.** Fully adjusted meta-risk estimates for categories of duration of use (stratified at the median duration of 16 years) of 11 pesticide chemical groups and 13 active ingredients and Hodgkin lymphoma diagnosed during follow-up in farmers and farm workers in three cohort studies from France, Norway, and the USA.

|  |  | Duration of exposure <16 years | | | |  | Duration of exposure ≥16 years | | | |  |
| --- | --- | --- | --- | --- | --- | --- | --- | --- | --- | --- | --- |
|  |  | N_exp_ | HR^a^ | 95% CI | I^2^ |  | N_exp_ | HR^a^ | 95% CI | I^2^ | p_trend_ |
| Organophosphate Insecticides |  |  |  |  |  |  | 24 | 0.23^b^ | 0.04-1.44 | 0% |  |
| Malathion |  | 19 | 0.60 | 0.15-2.35 | 52% |  | 22 | 0.35 | 0.06-2.12 | 46% | 0.30 |
| Parathion |  |  |  |  |  |  | 22 | 0.58^c^ | 0.09-3.68 | 48% |  |
| Carbamate Insecticides |  | 16 | 0.96 | 0.37-2.52 | 0% |  | 27 | 1.57 | 0.52-4.74 | 0% | 0.60 |
| Pirimicarb |  |  |  |  |  |  | 20 | 0.86 | 0.20-3.81 | 0% |  |
| Organochlorine Insecticides |  | 14 | 1.16 | 0.37-3.60 | 45% |  | 27 | 1.41 | 0.57-3.47 | 1% | 0.27 |
| DDT |  | 17 | 2.04^c^ | 0.80-5.20 | 0% |  |  |  |  |  |  |
| Lindane |  | 12 | 1.31 | 0.49-3.50 | 32% |  | 22 | 0.98^c^ | 0.36-2.67 | 0% | 0.45 |
| Pyrethroid Insecticides |  | 28 | 1.11 | 0.49-2.50 | 0% |  |  |  |  |  |  |
| Esfenvalerate |  | 21 | 1.89^c^ | 0.80-4.48 | 0% |  |  |  |  |  |  |
| (Phenyl) Urea Herbicides |  |  |  |  |  |  | 23 | 0.88^c^ | 0.26-3.03 | 0% |  |
| Linuron |  |  |  |  |  |  | 22 | 0.76^c^ | 0.23-2.56 | 0% |  |
| Chloroacetanilide Herbicides |  | 18 | 1.10^b^ | 0.32-3.80 | 57% |  |  |  |  |  |  |
| Phenoxy Herbicides |  |  |  |  |  |  | 33 | 1.25 | 0.48-3.22 | 0% |  |
| 2,4-D |  |  |  |  |  |  | 33 | 1.50 | 0.59-3.81 | 0% |  |
| MCPA |  |  |  |  |  |  | 24 | 1.88^c^ | 0.50-7.15 | 0% |  |
| Triazine Herbicides |  | 22 | 1.00 | 0.40-2.50 | 0% |  | 22 | 0.85 | 0.31-2.30 | 0% | 0.57 |
| Triazinone Herbicides |  | 18 | 0.77 | 0.34-1.73 | 0% |  |  |  |  |  |  |
| Metribuzin |  | 18 | 0.77 | 0.34-1.73 | 0% |  |  |  |  |  |  |
| Other Herbicides |  |  |  |  |  |  |  |  |  |  |  |
| Dicamba |  | 16 | 1.55 | 0.73-3.29 | 0% |  | 17 | 1.55 | 0.67-3.58 | 0% | 0.28 |
| Dithiocarbamate Fungicides |  |  |  |  |  |  | 22 | 1.42^c^ | 0.47-4.27 | 0% | 0.51 |
| Mancozeb |  |  |  |  |  |  | 21 | 1.37^c^ | 0.46-4.05 | 0% | 0.53 |
| Phthalimide Fungicides |  |  |  |  |  |  | 18 | 0.68^c^ | 0.19-2.52 | 0% | 0.82 |
| Captafol |  |  |  |  |  |  | 13 | 0.69^c^ | 0.24-1.99 | 0% | 0.54 |

Note: Only results based on at least two cohorts with 5 or more exposed cases each in the given duration category are reported. Pesticides that were investigated but did not meet the reporting rules were: dinitroaniline herbicides, thiocarbamate herbicides, arsenical pesticides and the active ingredients chlorpyrifos, dichlorvos, terbufos, aldicarb, carbaryl, carbofuran, deltamethrin, permethrin, isoproturon, alachlor, metolachlor, trifluralin, MCPP, butylate, EPTC, atrazine, simazine, glyphosate, thiram, and captan.

Abbreviations: meta-HR, meta-hazard ratio; CI, confidence interval; N_exp_, number of exposed cases; p_trend,_ p-value for trend; DDT, dichlorodiphenyltrichloroethane; 2,4-D, 2,4-dichlorophenoxyacetic acid; MCPA, 2-methyl-4-chlorophenoxyacetic acid; MCPP, methylchlorophenoxypropionic acid; EPTC, S-ethyl dipropylthiocarbamate.

^a^meta-HRs combining cohort-specific HR estimates adjusted for cohort-specific sets of confounders. AGRICAN: Cox regression adjusted for sex, livestock, retirement status, number of selected types of crops for which pesticide treatment personally applied, smoking status (current, former, or never); CNAP: Cox regression adjusted for sex, livestock, dichlorvos, aldicarb, lindane, DDT, deltamethrin, mancozeb, linuron, glyphosate; AHS: Cox regression adjusted for sex, state, livestock, terbufos, lindane, DDT, permethrin, dicamba, parathion, carbaryl, smoking status (current, former, or never).

^b^Meta-analysis based on CNAP and AHS only.

^c^Meta-analysis based on AGRICAN and CNAP only.
